# Supplementary material for: Predicting visual function by interpreting a neuronal wiring diagram
Source: Nature. 2024 Oct 2;634(8032):113–23. doi: 10.1038/s41586-024-07953-5 (PMC11446822; doi:10.1038/s41586-024-07953-5)
Supplement: Supplementary file 6 — CRF and ERF predictions for individual TmY4 and TmY9 cells. Analogous to Supplementary Data 3, but for TmY target types. Shown are the top four monosynaptic pathways, the strongest pathway passing through each of the top ten intermediary types (ranking from Extended Data Fig. 7), and the trisynaptic pathway Tm1–TmY–Dm3–TmY (see the section entitled Prediction of spatial normalization). [file 41586_2024_7953_MOESM6_ESM.zip › DataS4/TmY9q/720575940625050151.pdf]

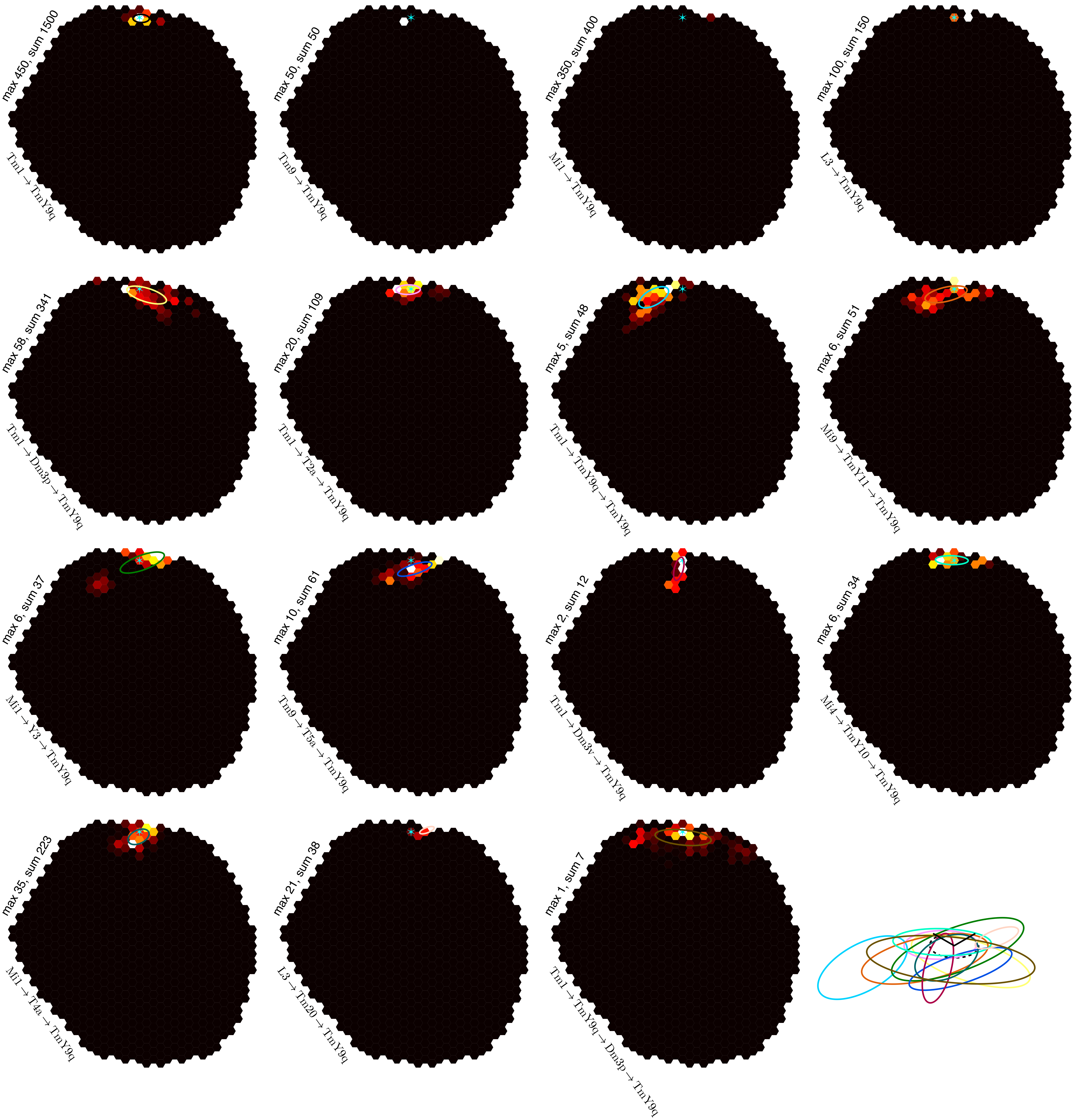

max 450, sum 1500
Tm1 → TmY9q
max 50, sum 50
Tm9 → TmY9q
max 350, sum 400
Mi1 → TmY9q
max 100, sum 150
L3 → TmY9q
max 58, sum 341
Tm1 → Dm3p → TmY9q
max 20, sum 109
Tm1 → T2a → TmY9q
max 5, sum 48
Tm1 → TmY9q → TmY9q
max 6, sum 51
Mi9 → TmY11 → TmY9q
max 6, sum 37
Mi1 → Y3 → TmY9q
max 10, sum 61
Tm9 → T5a → TmY9q
max 2, sum 12
Tm1 → Dm3v → TmY9q
max 6, sum 34
Mi4 → TmY10 → TmY9q
max 35, sum 223
Mi1 → T4a → TmY9q
max 21, sum 38
L3 → Tm20 → TmY9q
max 1, sum 7
Tm1 → TmY9q → Dm3p → TmY9q
